# Assessing local structural perturbations in proteins

**DOI:** 10.1186/1471-2105-6-226

**Published:** 2005-09-13

**Authors:** Martin A Lema, Julian Echave

**Affiliations:** 1 Universidad Nacional de Quilmes, Roque Sáenz Peña 180, B1876BXD Bernal, Buenos Aires, Argentina

## Abstract

**Background:**

Protein structure research often deals with the comparison of two or more structures of the same protein, for instance when handling alternative structure models for the same protein, point mutants, molecule movements, structure predictions, etc. Often the difference between structures is small, restricted to a local neighborhood, and buried in structural "noise" due to trivial differences resulting from experimental artifacts. In such cases, whole-structure comparisons by means of structure superposition may be unsatisfactory and researchers have to perform a tedious process of manually superposing different segments individually and/or use different frames of reference, chosen roughly by educated guessing.

**Results:**

We have developed an algorithm to compare local structural differences between alternative structures of the same protein. We have implemented the algorithm through a computer program that performs the numerical evaluation and allows inspecting visually the results of the structure comparison. We have tested the algorithm on different kinds of model systems. Here we present the algorithm and some results to illustrate its characteristics.

**Conclusion:**

This program may provide an insight into the local structural changes produced in a protein structure by different interactions or modifications. It is convenient for the general user and it can be applied to standard or specific tasks on protein structure research.

## Background

Localized perturbations in a protein structure can originate from point mutations, chemical modifications, interaction with other molecules, etc. Sometimes, it is necessary to compare alternative structures for the same protein sequence (e.g. different three-dimensional structure predictions, multiple models from NMR studies, etc.). To assess such protein structural perturbations, structures are usually compared in a detailed way, by looking at the position and orientation of individual atoms, residues, or secondary structures (for instance, see [1]). This approach is mandatory on case studies, because it leads to explain how modifications have changed the structure and function of a protein. However, this kind of comparison is usually done by superposing different particular structure elements individually and/or by using different protein-specific frames of reference, which are chosen according to the expertise and personal criteria of the researcher. This complicates establishing generalizations applicable to different proteins and the analysis of large numbers of cases. For such situations, quantitative measures of structural perturbation, such as the root mean square deviation (RMSd) or a derivative function [2-4], are used.

RMSd is a measure that is simple to calculate and to understand, it can be employed to establish comparisons through different structural families, it has been used very widely, and it is familiar to every researcher in the field. However, RMSd is usually referred to whole-molecule superpositions, so that it does not provide information on partial features, such as whether the perturbation is local or distributed throughout the whole molecule. This problem might be overcome by looking at the components of RMSd, the squared distances between pairs points compared. However, there is a problem with this approach, inherent to whole-molecule superpositions: portions of the structure with little or no perturbation may be badly superposed in order to improve the superposition of those portions with important distortions.

Several authors have developed effective methods for sequence alignment based on local structural features [4-13]. Most of these methods do not work with neighborhoods of equal and fixed size, or the local zones are not equally distributed along the molecule. This is not a problem for the task of structural alignment because, once a local zone is defined, the goal is usually to optimize whatever measure is used inside that zone. On the other hand, for the detection and description of structural alterations it is essential that all local zones have the same size, in order to compare among them on a uniform basis, therefore allowing the reliable identification of zones with a perturbation that is significantly different from the average. Moreover, these methods refer (and restrict) their local zones to a secondary structure element or a window of residues along the sequence. These are one-dimensional boundaries, which while being convenient for aligning sequences, are poor to examine three-dimensional structure perturbations, which may involve atoms from residues that are not close in the linear sequence, whose interactions are thus neglected.

Specifically for the recognition and measure of local structural alterations, we propose that it is more suitable to compare structures on a uniform and residue-based approach, and by delimiting the neighborhood of each residue just in terms of distances. As a result, we conceive a unit of comparison integrated by a residue and the group of adjacent atoms within a fixed radius. Therefore, we have developed an algorithm to quantify the degree of structural alteration in the local neighborhood of each residue, when comparing two or more structures, and the means of exploiting this measure not only analytically but also visually.

## Implementation

COLORES (Comparison of LOcal Residue Environment Structures) is a program that compares two or more protein structures, by performing an assessment of the local structural alteration in the neighborhood of each residue. The input is a set of protein structure files in PDB (Protein Data Bank) format, a sequence alignment between those structures in GDE format (Genetic Data Environment, Steven Smith, 1994, Version 2.3), and a set of user choices described below. The program generates a log file containing detailed information of each local comparison, a data file containing summaries per alignment position, a structure (PDB-formatted) file and a script file for the RASMOL [14] program, which allows the user to inspect the results visually. COLORES automatically invokes RASMOL to show the results after its job is done.

For each position in the alignment, COLORES calculates two scores, described next:

### Truly local score

**Figure 1:**
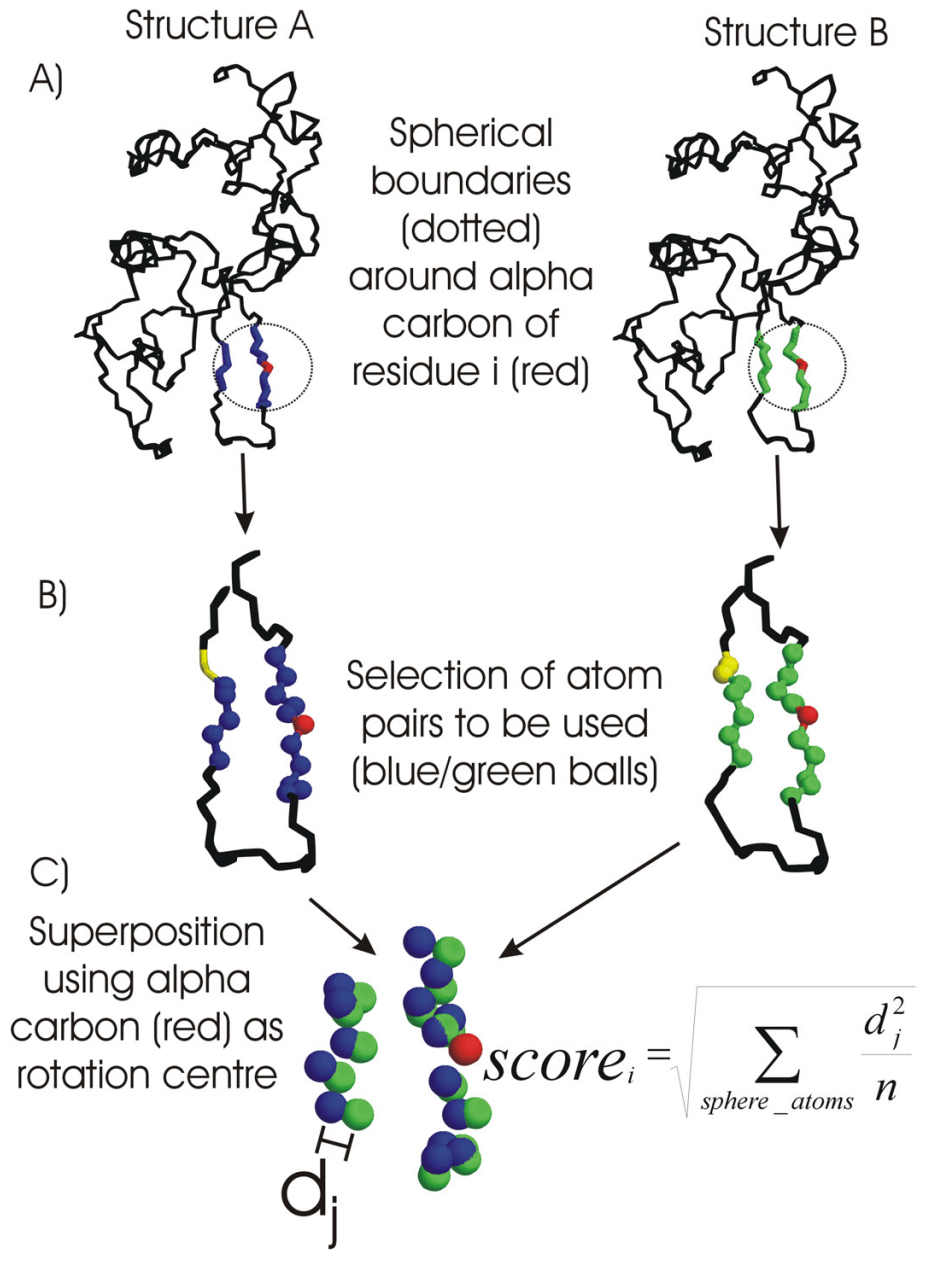
**Calculation of the *truly local *score. **This figure explains how the truly-local score is calculated for a single residue in a pairwise comparison (using the default parameters for the program). Previously, a user-provided alignment allows establishing which residues and atoms of one structure are equivalent to which ones in the other. A) On each structure to be compared, a sphere is considered around the alpha carbon atom (red) of the residues corresponding to the alignment position whose truly-local score will be calculated. Two lists including all the backbone atoms falling inside each sphere are prepared (atoms which belong to one list are rendered blue, while the atoms which belong to the other are rendered green). B) Lists are compared to find equivalent atoms. The atoms that were inside one or another sphere are now rendered as balls. The color of atom pairs whose members were one of them inside one sphere and the other outside has been changed to yellow, and these pairs are dismissed from the comparison. C) The remaining atom pairs (whose members were both inside their respective sphere) are taken as a group of fixed points. Then, the alpha carbon atoms being the former centers of the spheres are superposed, and the system is rotated until the root mean square of the atom pair distances reaches a minimum. This minimum value is the score for the residue considered. All the process is iterated for each residue along the sequence. Variations and alternatives on the procedure are discussed in the main text.

The algorithm compares protein structures on a residue-by-residue basis. It calculates a score for each alignment position having no gaps on either sequence. The calculation is performed as described next:

For each structure (see Figure 1A), a sphere whose radius is chosen by the user is defined around the residue under consideration. These spheres can be centered either at the alpha carbon atom or at the center of mass of their respective residues, according to the user's choice.

Then, two lists are prepared containing all atoms inside each sphere. There are three options regarding the kind of atoms to be included (this is again a user choice defined as "eligible atoms"): all heavy (non-hydrogen) atoms, backbone atoms, or alpha carbon atoms.

Next (see Figure 1B), the algorithm selects which atom pairs will actually be used for calculations (the pairs of equivalent atoms are inferred from the residue equivalence provided in the alignment file). The user option at this stage is to use only atom pairs whose members are both inside their corresponding spheres (i.e. the intersection of the two lists or "intersection set"), or pairs with at least one member inside one of the spheres (the "union set").

After selecting the atom pairs, the sets of points (corresponding to the relative locations of the atom centers) are placed in the same coordinate system, with the centers of both spheres at the origin (see Figure 1C).

Finally, the quaternion method [15] is used to find the rotation of one set of points around the origin that minimizes the RMSd between the two sets of points. The final RMSd value is used as a measure of the perturbation for the region surrounding the residue considered. We will refer to it as the "truly-local score".

In this way a score value is obtained per each non-gapped position of the alignment, for the comparison of two structures. If there are more than two structures to analyze, every one of them is iteratively compared against the rest, and the final score per alignment position is the average of scores obtained from all the pairwise comparisons.

**Heuristic penalties: **mutations or the use of the "intersection set" (see above) can result in unpaired atoms. In this case, the user can choose either to ignore these atoms or to introduce a heuristic penalty to account for their presence in one neighbor list but not in the other. It can be argued that these unpaired atoms actually reflect a difference in a residue neighborhood from one protein to the other, which should be accounted for. The penalty function is largest when the distance from the unpaired atom to the center of the sphere is zero, and it decreases smoothly to zero when this distance is equal to the sphere radius:



where **p**_max _is a maximum penalty value (set by the user), **r **stands for the sphere radius, and **d**_u _is the distance from the unpaired atom to the center of the sphere. The score formula, introduced in Figure 1, is modified to account for the penalty in this way:



where *n *is the number of paired atoms and *m *is the number of unpaired atoms.

### Pseudo-local score

COLORES also calculates an alternative score, which does not make use of a sphere nor any "neighborhood" concept. A structural superposition of the two whole structures is performed as usual: the complete structures are used, the rotations are centered at the center of mass of the structures, and the RMSd for the whole structure is minimized using the quaternion method.

Then, the distance between equivalent alpha carbon atom pairs (from residues corresponding to the same alignment position) is taken as an alternative "pseudo-local" score. This measure is, in some way, the one used when a researcher superposes two structures and visually analyses the distance between the backbones. This score is not presented as a novelty but as a reference of one of the current ways of looking for "local" structure alteration or conservation, and to show the significant improvement represented by the truly-local score. The pseudo-local score suffers from the drawbacks mentioned before for whole-molecule superpositions, i.e. that portions of the structure with little or no perturbation are superposed badly in order to improve the superposition of those portions with important perturbations.

### Output visualization

Along with the detailed log file and the data file containing the scores per alignment position, a structure file is produced whose atom coordinates can be selected to be either:

a) The coordinates of the first protein of the alignment.

b) Average coordinates corresponding to each position: the coordinates of equivalent backbone atoms are averaged after their structures have been superposed. The average structure is only created for visualization purposes; it is not used for the calculation of the scores.

The truly-local and/or the pseudo local score are also saved in the structure file, in the data column corresponding to the b-factor (if both scores are selected for display, two identical structures are saved, each one of them with a different score in the b-factor column). This allows displaying the scores on the structure, by employing different colors and different backbone widths; both means of visualization are used simultaneously to aid the visual inspection of the results. This also allows the user to modify further this display by using one or the other property to show a different specific feature, while still showing the COLORES scores with the remaining property. The three-dimensional presentation of numerical scores and other information as colors or shapes has proven to be a powerful tool for analyzing this kind of data (e.g. see [16]), because it allows to appreciate spatial relationships that are not so evident in a bi-dimensional graph.

A script file for RASMOL is created in order to launch the display automatically, and to spare the user the need to learn how to configure the program (many other programs can also perform a similar display if properly set up by the user).

For a better display, the scores saved in the structure file differ from the original ones (saved in the data file), in the sense that they are internally normalized and corrected to account for gapped positions (as fully explained in the program documentation).

## Results and discussion

We have used COLORES to analyze examples taken from different works on protein structure research. Here we provide a detailed description of two cases, for which we compare COLORES with other structural comparison software. Additional examples are available at the COLORES webpage.

Unless stated otherwise, in the examples provided we have used default parameters for the program, which can be found (and modified) in a key file accompanying the executable files distribution. The default values for the more significant options are: (a) the sphere has a radius of 10 A and (b) it is centered at the alpha carbon atom of the residues, (c) the atoms eligible for comparison are all backbone atoms, (d) only atom pairs whose members are inside the spheres in both structures are included in the comparison ("intersection set"), and (e) there is no heuristic penalty to account for unpaired atoms.

Values near 10 A are usually used to define a limit in comparable studies, were the goal is also to reduce the scope of a calculation to the relevant neighborhood, like the cut-offs for atomic interacting forces in molecular mechanics calculations, or the spatial boundary for the Ooi's number [17]. The use of backbone atoms is an appropriate and popular choice for protein structure comparison, although in some cases the alternative possibilities are better suited, for instance when comparing prediction models that are made only of alpha carbon atoms traces, or for the analysis of two very similar structures that may require using all heavy atoms. Regarding the remaining choices, which are considering only atom pairs whose members are inside the spheres in both structures and not using heuristic penalties to account for unpaired atoms, both obey to the criteria of keeping the calculation as simple as possible, in order to make it more transparent to the novel user and avoiding the introduction of additional parameters.

This is a "first approach" and all-purpose set of choices that the user, after an initial test run, in some cases may change to address better his/her specific protein model, goals, and personal criteria.

### Protein structure prediction

The assessment of protein structure predictions (models) is an area where our algorithm can make a significant contribution. A three-dimensional structural model of a protein is a powerful asset in the investigation of its biological function (for instance, see [18,19]), but producing such a model through experimental determinations is not always easy or even possible. As a result, powerful programs to produce theoretical predictions are being developed (for example, ROSETTA [20,21]). The different prediction tools are contrasted in the Critical Assessment of Protein Structure Prediction (CASP) a community-wide experiment where sequences of proteins, whose experimental structures will be released soon, are communicated to groups working in the field so that they can make their predictions [22,23]. Original algorithms have been developed and used for the analysis of CASP predictions [24,25]. COLORES is also a valuable tool for this purpose, because it can help to compare both visually and analytically different predictions for a given target.

We will compare the results from COLORES and the RMS/Coverage method. The latter is adequately explained in references [25,26]; in brief, it reports, for a given fraction of the protein residues (coverage), which combination of residues exhibits the best superposition.

The CASP prediction T0030AB807 is analyzed using this method in [25]. The main conclusions of the RMS/Coverage analysis are:

a) For superpositions of up to four residues, the zone around residues 20-23 exhibits the best superposition.

b) For superpositions comprising between 5 and 18 residues, the best superposition primarily involves residues in a hairpin centered on residues 48-49 (from 11 to 18 residues, however, a separated short stretch around residue 26 is also included). For superpositions involving 19 residues or more, a different set of residues comprise the best superposition, this leads to the conclusion that the hairpin structure is well predicted locally but not with respect to the rest of the structure.

c) For superpositions involving 19 residues or more, residue stretches corresponding to four different protein zones integrate the best superposition set. All these stretches grow simultaneously along with the increase in coverage.

**Figure 2:**
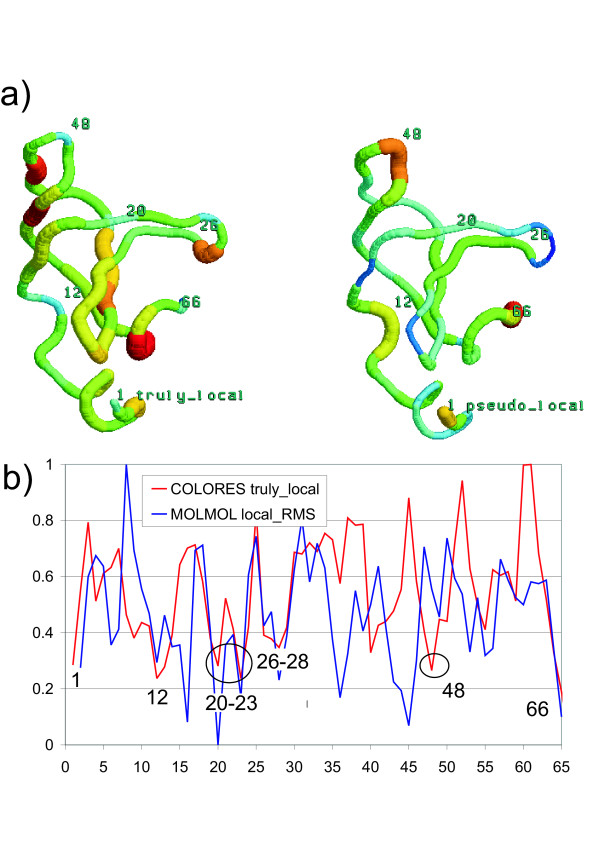
**Assessment of a protein structure prediction. **(A) COLORES comparison of CASP prediction T0030AB807 (coordinates from [22]) against an average structure from a set of 15 NMR experimental structures (PDB code: 1FGP) of the target sequence. The truly-local score is displayed on the left side and the pseudo-local score on the right side. The higher scores (higher local structural differences) are represented by a thicker backbone trace and colors closer to red in the spectrum, while lower scores are represented by a thinner trace and colors closer to blue. (B) The profile of different scores along the protein sequence: COLORES "truly-local" score (red) using standard values and MOLMOL "local RMS" score (blue). Scores have been normalized for a better contrast.

In Figure 2 we show the COLORES results from comparing the predicted structure with the experimentally determined target structure. It can be seen that the residue neighborhoods with smallest truly-local scores are those around residues 1, 12, 48, 26-28, 20-23, and 66; three of them correspond to the zones reported in the RMS/Coverage results, and three are new. Zones found by COLORES but not by RMS/Coverage are those still well predicted locally but ranking second to other with the same coverage (because RMS/Coverage only reports the best).

There are differences in the way that residues are included in the lists for the two programs, and this is reflected in the output. For instance, for the loop around residues 48-49, RMS coverage shows most of it in the best coverage list, but COLORES just marks a low score for residue 48. This is because the neighborhood of residue 48 is composed almost entirely of residues in the loop, while the neighborhoods of the remaining residues of the loop include atoms from other parts of the protein. This means that residue 48 has a relatively better predicted neighborhood, while the neighborhoods of the other residues in the loop include both well predicted and poorly predicted protein regions.

RMS/Coverage and COLORES are similar in one feature: they report results from the superposition of atoms that belong to a list of equivalent atoms. The main difference is that RMS/Coverage atom lists are made from a combination of residues taken from any part of the protein, provided that they exhibits the lower RMSd after superposition; while COLORES lists belong to atoms surrounding a certain residue. The other difference is that if two different protein zones of a similar size are especially well predicted, RMS/Coverage will report just the best superposition, while COLORES will allow noticing the two of them due to their low score.

RMS/Coverage can indicate when a part of the protein is well predicted locally and not with respect to the rest of the protein, because a single zone will have the best RMSd at low coverages but not at larger coverages. On the other hand, the truly-local score of COLORES can show multiple zones that have been well predicted locally, but it does not indicate how these zones have been predicted in the context of the rest of the structure. This can be alleviated partially by looking at the pseudo-local score, as it is based on a whole-molecule superposition. When a neighborhood is well predicted locally (i.e. it has a low truly-local score), if it has a high pseudo-local score it can also be concluded that it is badly predicted with respect to the rest of the structure.

The RMS/Coverage method sometimes reports a single "best superposition" for a given coverage, which if formed by unrelated structure patches (e.g. the two zones around residues 26 and 48 at a coverage of 18 residues, for the present example). These zones are not sequentially close, neither are they near in three-dimensional space, nor belong to the same secondary structure element. What can be deduced from the fact that, when arbitrarily grouped and separated from the rest of the structure, these two structure patches superpose well? Since COLORES reports results of a zone that represents a spatial neighborhood of a residue, its unit of comparison always has an objective interpretation. Besides, when reporting one of these artificial merges, RMS/Coverage may overlook a zone with more structural significance and a good local superposition that does not have the single best RMSd for the same coverage level; this is not likely to happen when using COLORES.

Summing up, COLORES offers two main advantages:

- When two or more zones of similar size have been well predicted, COLORES reports all of them simultaneously.

- COLORES reports a definite score for each residue. In addition, the scores correspond to a protein zone that has a significant meaning (a fixed-size three-dimensional neighborhood of a residue).

A secondary advantage of COLORES versus RMS/Coverage is that COLORES is actually available for download and use, while RMS/Coverage is not available as a software application (just its results on the analysis of past CASP predictions).

It is also worthwhile to compare COLORES with MOLMOL [27], which is a software widely used for structure visualization and comparison. To compare two or more structures, MOLMOL calculates a "local RMSd" by iteratively superposing all combinations of three contiguous residues, and then assigning the RMSd value to the middle one. MOLMOL also calculates a score named "average global displacements" which is the same as the pseudo-local score calculated by COLORES.

When the MOLMOL local RMSd is calculated for our present example (see figure 2b), it can be seen that it detects several three-residue-long windows of low RMSd, being the one of lowest value around residue 20 (as reported independently by the RMS-Coverage method at a coverage level of three residues). But all the other zones which are well predicted and detected by the other methods (like the loop around residue 48) cannot be found using MOLMOL local RMSd. This is because RMS/Coverage and COLORES can take into account bigger sets of atoms. Therefore, MOLMOL shares with COLORES the property of reporting secondary well-predicted zones, but it is restricted to analyze only very low linear stretches of three residues. The idea behind COLORES is to enclose a significant neighborhood, big enough to include atoms that do not necessarily belong to very close and sequentially connected residues.

### Macromolecular movements

We have found that COLORES is also especially suited to analyze concerted molecular movements that involve a hinge or shear movement of an entire protein domain [28]. In these cases, standard "whole molecule" superposition is doomed to fail, because there is no global similarity between the two related structures. In contrast, local superposition can sharply differentiate which zones have maintained its local structure and where the structural alteration (allowing the movement) has occurred. We have tested the program against several examples from the *Database of Macromolecular Movements *[29,30]. Here we detail the example of the calmodulin protein.

The unligated form of Calmodulin is composed of two globular domains connected by a long helix. The protein can bind peptide helices by closing the two domains in a hinge motion, which breaks the long helix in two minor helices with a strand in between.

**Figure 3:**
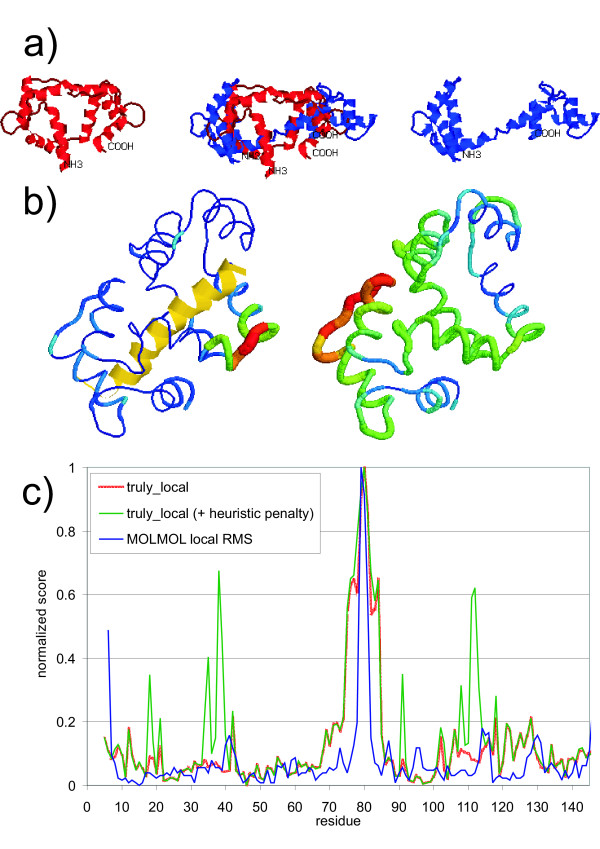
**Macromolecular movement of calmodulin. **A) The closed state of calmodulin (left, PDB code: 1MXE), the open state (right, PDB code: 3CLN) and a standard whole molecule superposition (middle). B) The truly-local and pseudo-local COLORES scores displayed on the closed form. On the left: the local score showing two structurally well-conserved regions (thin) and one disturbed hinge (thick); the yellow helix is the bonded peptide. On the right side: the global score, which fails to identify these key structural features (displayed on the same structure, peptide is omitted). C) The profile of different (normalized) scores along the protein sequence: COLORES "truly-local" score (red) using standard values, COLORES "truly-local" score using heuristic penalties (green), and MOLMOL "local RMS" (blue).

The standard whole molecule superposition displayed in figure 3a clearly shows the inadequacy of this approach to differentiate portions of the structure with little or no perturbation (i.e. the globular domains) from the connecting helix, which does suffer important perturbations. This is also reflected by the profile of the pseudo-local score on figure 3b (right). On the contrary, the truly-local score shown in figure 3b (left) clearly discriminates these zones.

This example shows that our algorithm may contribute to discriminate the unaltered domains from hinge or otherwise structurally altered regions, and therefore to detect evidence of this kind of molecular movements. Moreover, this is achieved without employing any particular knowledge about the protein function or structure. Considering that the number of existing protein structures increases exponentially, and concurrently more structures belonging to the same protein but determined (or predicted) under different circumstances are available, COLORES may help to find and even to automate the process of molecular movement detection as a complement of other tools like the Sieve-Fit Procedure [31] or the Multiple Linkage Clustering [32].

Regarding the comparison with other software, as a program for calculating RMS/Coverage is not available, we cannot provide an actual analysis. Nevertheless, it is evident that it would report a single globular domain having the best coverage, but not both of them simultaneously. In Figure 3c we compare COLORES with MOLMOL. It can be seen that MOLMOL local RMSd score reports three highly perturbed residues (79-81), while COLORES reports the 75-84 residue stretch. MOLMOL only detects coarse main chain alterations from one structure to the other, in the center of the hinge where the long helix is broken. On the other hand, COLORES also incorporates the changes in the neighborhood of all the residues along the hinge, so it allows defining this region entirely. Independently, the hinge region has been defined by the residues with the largest torsion angle differences (reported to be located in residues 72 to 82) [30], confirming the better sensitivity of the COLORES analysis.

It is interesting to note, before leaving this example, that when a heuristic penalty is introduced in the truly local score (or when the "union set" is selected), the output is significantly changed. Two zones in each domain increase their score, showing that the neighborhood of the corresponding residues was actually changed; this is due to atoms from the opposite domain, which moved from a nearby position in the closed form to a long distance in the open form.

## Conclusion

The comparison of protein structures is an established tool for investigating biological function, macromolecular structure, protein evolution, etc. The superposition of entire structures is the standard approach to initiate this analysis but, in some cases, it can produce misleading results.

A local approach for structural comparison can lead to a better insight and discrimination of perturbed against unchanged portions of the structure. Local comparison has been used mostly in the area of structure-based sequence alignment, by employing approaches suited for that particular purpose, but not for the general description of how each zone of a protein changes between two or more structures.

We have developed an algorithm to describe local perturbations of protein structures in quantitative rather than descriptive terms. The method is applicable to any situation and its results are comparable between very different cases. Besides, a means of analyzing its results visually is provided by the program that implements the algorithm; this is a valuable asset in order to interpret three-dimensional results. The analysis of structural perturbations is not a task that can be done only with numbers and tables; sometimes it is necessary to use visual inspection to discriminate what is relevant, and to search for relationships between structural conservation/perturbation and (bio)chemical modifications, biological function, changes in the crystal contacts, etc. We have compared our results with other existing software to show that the present method offers a useful alternative for the analysis of protein structures.

The examples provided in this article, and others available in the software webpage, show that the program can be used easily to perform standard tasks on protein structural research, like:

a) On experimentally generated structures: the comparison of mutant and wild type proteins, the comparison of enzymes in open/close conformation (or proteins with/without ligand), the comparison of protein structures determined from different sources, and the comparison of structurally similar proteins in general.

b) On computationally generated structures: the assessment of structure alterations after molecular mechanics manipulations like minimization, molecular dynamics simulation, etc.; the comparison of different three-dimensional structure predictions either among them or against the target structure.

COLORES can also be used in less standard applications, like the definition of evolutionarily conserved cores, or the identification of zones implicated in macromolecular movements.

COLORES provides numerical results that can be used for quantitative purposes, and are comparable even across different protein structural families. Its scores are also displayed over the structures under study, in a way that can be interpreted quickly and easily. These characteristics make it both suitable for "first glance" purposes when approaching to a novel system under study, and for more specific analytic tasks. Its local approach facilitates finding which zones have been perturbed and how much, particularly when using the truly-local score.

It is important to note that, in its present implementation, COLORES is only able to calculate trivial alignments for pairwise comparisons, when they involve proteins having the same sequence length, and at most a few point mutations. For comparisons involving multiple structures and/or notoriously different sequences, COLORES does not perform nor improve an alignment between the structures under analysis; but instead it requires the alignment as an input, as described before. We are aware that our comparison method could be developed further as a structural alignment algorithm, and although we may explore that possibility in the future, actually a variety of quite well-developed options are already available for performing the alignment of two protein structures [33,34].

Finally, we would like to consider another possible extension of the method. It has been established that cutoff distances for physical potentials are useful to speed molecular mechanics simulations [35]. In these cases, a spherical limit defines a neighborhood of atoms that effectively interact with the atom whose energetic contribution is being calculated. An energetic local score could be calculated from the difference between single point calculations for all the atoms inside the spheres used in COLORES. This would complement the present geometry-based local score with an energetic one. This, in turn may be useful to assess if a geometrical perturbation has an energetic counterpart. For instance, a conservative mutation may only produce an increment in the geometric score but not in the energetic score, while a non-conservative mutation may have an impact on both.

## Availability and requirements

**Project name: **COLORES software

**Project home page: **. The files used in the examples described in this paper, among others, can also be downloaded from that Internet address, in order to analyze them interactively.

**Operating system(s): **Executable files tested for Windows or Linux are available. The source code was written to be platform independent, and it is also available.

**Programming language: **ANSI standard C.

**Other requirements: **A software capable of showing temperature factors on protein structures as a scale of variable color and backbone width. It is not required for COLORES to run (nor to analyze results analytically), but just in order to check results graphically. It is convenient to use RASMOL, since COLORES automatically calls the program and execute the proper commands for a better display, with no need of user intervention. Other software (e.g. MOLMOL [27]) can be used instead, but in this case, the user must write the appropriate script file or commands.

**License: **Free for academic purposes.

**Any restrictions to use by non-academics: **contact the authors.

## Authors' contributions

Both authors developed the method. ML developed and tested the software under the supervision of JE. ML wrote the manuscript and JE edited the final version.

## References

[B1] Paoli M, Liddington R, Jeremy T, Wilkinson A, Dodson G (1996). Crystal Structure of T State Haemoglobin with Oxygen Bound At All Four Haems. J Mol Biol.

[B2] Bruschweiler R (2003). Efficient RMSd Measures for the Comparison of Two Molecular Ensembles. Proteins.

[B3] Jewett AI, Huang CC, Ferrin TE (2003). Minrms: an Efficient Algorithm for Determining Protein Structure Similarity Using Root-Mean-Squared-Distance. Bioinformatics.

[B4] Yang AS, Honig B (2000). An integrated approach to the analysis and modeling of protein sequences and structures. I. Protein structural alignment and a quantitative measure for protein structural distance. J Mol Biol.

[B5] Kobayashi N, Nobuhiro G (1997). A method to search for similar protein local structures at ligand-binding sites and its application to adenine recognition. Eur Biophys J.

[B6] Koehl P (2001). Protein structure similarities. Curr Opin Struct Biol.

[B7] Leibowitz N, Nussinov R, Wolfson HJ (2001). MUSTA - a general, efficient, automated method for multiple structure alignment and detection of common motifs: application to proteins. J Comput Biol.

[B8] Leluk J, Konieczny L, Roterman I (2003). Search for structural similarity in proteins. Bioinformatics.

[B9] Lehtonen JV, Denessiouk K, May AC, Johnson MS (1999). Finding local structural similarities among families of unrelated protein structures: a generic non-linear alignment algorithm. Proteins.

[B10] Ochagavia ME, Richelle J, Wodak SJ (2002). Advanced pairwise structure alignments of proteins and analysis of conformational changes. Bioinformatics.

[B11] Orengo CA, Taylor WR (1993). A Local Alignment Method for Protein-Structure Motifs. J Mol Biol.

[B12] Szustakowski JD, Weng Z (2000). Protein structure alignment using a genetic algorithm. Proteins.

[B13] Ye Y, Jaroszewski L, Li W, Godzik A (2003). A segment alignment approach to protein comparison. Bioinformatics.

[B14] Bernstein HJ (2000). Recent changes to RasMol, recombining the variants. Trends Biochem Sci.

[B15] Kearsley SK (1989). On the Orthogonal Transformation Used for Structural Comparisons. Acta Crystallogr A.

[B16] Altman RB, Hughes C, Gerstein M (1995). Methods for displaying macromolecular structural uncertainty: application to the globins. J Mol Graph.

[B17] Nishikawa K, Ooi T (1986). Radial locations of amino-acid residues in a globular protein – correlation with the sequence. J Biochem.

[B18] Lee SJ, Sekimoto T, Yamashita E, Nagoshi E, Nakagawa A, Imamoto N, Yoshimura M, Sakai H, Chong KT, Tsukihara T, Yoneda Y (2003). The Structure of Importin-β Bound to SREBP-2: Nuclear Import of a Transcription Factor. Science.

[B19] Wedemayer GJ, Patten PA, Wang LH, Schultz PG, Stevens RC (1997). Structural insights into the evolution of an antibody combining site. Science.

[B20] Bradley P, Chivian D, Meiler J, Misura KM, Rohl CA, Schief WR, Wedemeyer WJ, Schueler-Furman O, Murphy P, Schonbrun J, Strauss CE, Baker D (2003). Rosetta predictions in CASP5: successes, failures, and prospects for complete automation. Proteins.

[B21] Kuhlman B, Dantas G, Ireton GC, Varani G, Stoddard BL, Baker D (2003). Design of a novel globular protein fold with atomic-level accuracy. Science.

[B22] Tramontano A, Morea V (2003). Assessment of Homology-Based Predictions in CASP5. Proteins.

[B23] Protein Structure Prediction Center (CASP website). http://predictioncenter.llnl.gov.

[B24] Zemla A (2003). LGA: a method for finding 3D similarities in protein structures. Nucleic Acids Res.

[B25] Hubbard TJ (1999). RMS/coverage graphs: a qualitative method for comparing three-dimensional protein structure predictions. Proteins.

[B26] RMS/Coverage method website. http://predictioncenter.llnl.gov/casp3/results/th/method.html.

[B27] Koradi R, Billeter M, Wüthrich K (1996). MOLMOL: A program for display and analysis of macromolecular structures. J Mol Graph.

[B28] Krebs WG, Tsai J, Alexandrov V, Junker J, Jansen R, Gerstein M (2003). Tools and databases to analyze protein flexibility; approaches to mapping implied features onto sequences. Methods Enzymol.

[B29] Database of Macromolecular Movements. http://molmovdb.mbb.yale.edu/.

[B30] Echols N, Milburn D, Gerstein M (2003). MolMovDB: analysis and visualization of conformational change and structural flexibility. Nucleic Acids Res.

[B31] Gerstein M, Chothia CH (1991). Analysis of protein loop closure: two types of hinges produce one motion in lactate dehydrogenase. J Mol Biol.

[B32] Boutonnet NS, Rooman MJ, Wodak SJ (1995). Automatic analysis of protein conformational changes by multiple linkage clustering. J Mol Biol.

[B33] Barton GJ (1998). Protein Sequence Alignment Techniques. Acta Crystallogr D.

[B34] Carugo O, Pongor S (2002). Recent progress in protein 3D structure comparison. Curr Protein Pept Sc.

[B35] McCammon JA, Harvey SC (1987). Dynamics of proteins and Nucleic Acids.

